# EAAT2 Expression in the Hippocampus, Subiculum, Entorhinal Cortex and Superior Temporal Gyrus in Alzheimer’s Disease

**DOI:** 10.3389/fncel.2021.702824

**Published:** 2021-09-13

**Authors:** Jason H. Y. Yeung, Thulani H. Palpagama, Oliver W. G. Wood, Clinton Turner, Henry J. Waldvogel, Richard L. M. Faull, Andrea Kwakowsky

**Affiliations:** ^1^Centre for Brain Research, Department of Anatomy and Medical Imaging, Faculty of Medical and Health Sciences, University of Auckland, Auckland, New Zealand; ^2^Department of Anatomical Pathology, LabPlus, Auckland City Hospital, Auckland, New Zealand

**Keywords:** glutamate transporter, EAAT2, hippocampus, subiculum, entorhinal cortex, superior temporal gyrus, Alzheimer’s disease

## Abstract

Alzheimer’s disease (AD) is a neuropathological disorder characterized by the presence and accumulation of amyloid-beta plaques and neurofibrillary tangles. Glutamate dysregulation and the concept of glutamatergic excitotoxicity have been frequently described in the pathogenesis of a variety of neurodegenerative disorders and are postulated to play a major role in the progression of AD. In particular, alterations in homeostatic mechanisms, such as glutamate uptake, have been implicated in AD. An association with excitatory amino acid transporter 2 (EAAT2), the main glutamate uptake transporter, dysfunction has also been described. Several animal and few human studies examined EAAT2 expression in multiple brain regions in AD but studies of the hippocampus, the most severely affected brain region, are scarce. Therefore, this study aims to assess alterations in the expression of EAAT2 qualitatively and quantitatively through DAB immunohistochemistry (IHC) and immunofluorescence within the hippocampus, subiculum, entorhinal cortex, and superior temporal gyrus (STG) regions, between human AD and control cases. Although no significant EAAT2 density changes were observed between control and AD cases, there appeared to be increased transporter expression most likely localized to fine astrocytic branches in the neuropil as seen on both DAB IHC and immunofluorescence. Therefore, individual astrocytes are not outlined by EAAT2 staining and are not easily recognizable in the CA1–3 and dentate gyrus regions of AD cases, but the altered expression patterns observed between AD and control hippocampal cases could indicate alterations in glutamate recycling and potentially disturbed glutamatergic homeostasis. In conclusion, no significant EAAT2 density changes were found between control and AD cases, but the observed spatial differences in transporter expression and their functional significance will have to be further explored.

## Introduction

Alzheimer’s disease (AD) is the most common cause of dementia worldwide and is linked with a decline in cognitive function, behavior, and memory (McKhann et al., 1984). Alongside the predominant tau and beta-amyloid (Aβ) hypotheses, glutamatergic dysfunction has also been implicated in the pathogenesis of AD, with a significant effect on neuronal functioning and survival. Glutamatergic dysfunction is mediated through a range of mechanisms, including Aβ binding to glutamate receptors, tau tethering to intrinsic cytoskeletal proteins resulting in overactivation of receptors, and the internalization of glutamate transporters leading to glutamate accumulation in the synaptic and extrasynaptic space (Butterfield and Pocernich, 2003).

Glutamate is an endogenous amino acid with many physiological functions, including the synthesis of a variety of micro- and macro- compounds (Erecinska and Silver, 1990). In the central nervous system (CNS), glutamate serves three main functions: as an excitatory neurotransmitter, as the precursor molecule in the synthesis of γ-aminobutyric acid (GABA), the main inhibitory neurotransmitter in the CNS (Erecinska and Silver, 1990), and as a fuel for mitochondrial metabolism (Dienel, 2013; McKenna et al., 2016). It plays a central role in the regular functioning of cognition, memory, and learning, as well as overall normal brain performance (Fonnum, 1984). Homeostasis is maintained through efficient reuptake of glutamate by glial transporters, with most of the released neurotransmitters successfully contained within the local tripartite synaptic area (Vizi, 2000; Butterfield and Pocernich, 2003). Reuptake is predominantly astrocytic, resulting in a unidirectional glutamate shift from neurons to astrocytes, where glutamate is metabolized into glutamine *via* glutamine synthase. This flux of glutamate from neurons to astrocytes is restored through the transfer of glutamine from astrocytes back into neurons (Bak et al., 2006). The majority of glutamate uptake is through excitatory amino acid transporter 2 (EAAT2; rat GLT-1), which displays predominantly astrocytic expression patterns (Sheldon and Robinson, 2007; Rimmele and Rosenberg, 2016). Due to its tight regulation in normal physiology, the disruption of glutamate homeostasis as a mechanism for neuronal damage is one of the leading hypotheses implicating the glutamatergic system in AD pathogenesis.

The excitatory amino acid transporters (EAATs) are responsible for the uptake of glutamate from the extracellular space after its release from the presynaptic neuron (Purves, 2012). EAAT expression is highly brain-region specific. EAAT1 is highly expressed within the cerebellum (Storck et al., 1992) and plays an important role in neurodevelopment (Furuta et al., 1997b). EAAT2 is the primary glutamate transporter, responsible for ~95% of glutamate uptake, and is widely expressed on astrocytes throughout the CNS (Rothstein et al., 1994; Vandenberg and Ryan, 2013). EAAT3 is present mainly in post-synaptic neurons throughout the brain and is highly expressed within the hippocampus, cerebellum, and basal ganglia (Rothstein et al., 1994). EAAT4 and 5 are chloride channels (Fairman and Amara, 1999) with weak glutamate transporter properties (Gameiro et al., 2011) and are present in the cerebellum (Furuta et al., 1997a) and retina respectively (Arriza et al., 1997). EAAT1–3 share similar mechanisms of glutamate transport. In each cycle, one glutamate molecule is cotransported with three Na^+^ ions and one H^+^ ion, with one K^+^ ion transported in the opposite direction (Kanai et al., 1995; Wadiche et al., 1995).

Alterations in the regulation and expression of EAAT2 have been reported in both acute neurological conditions (Torp et al., 1995) and chronic neurodegenerative disorders (Rothstein et al., 1995; Li et al., 1997; Munch et al., 2002). Impairment of EAATs has been reported in AD, with studies observing a reduction in glutamate transporter capacity and selective loss of vesicular glutamate transporters with a concomitant rise in extracellular glutamate concentration (Li et al., 1997; Gu et al., 2004). This has been attributed partly to damage by reactive oxygen species and products of lipid peroxidation (Danysz and Parsons, 2012). Reversal of glutamate uptake has also been shown through the Aβ-mediated release of glutamate from microglia (Noda et al., 1999). This results in glutamate excitotoxicity, with glutamate diffusing into extrasynaptic areas and activating extrasynaptic receptors, notably N-methyl-D-aspartate (NMDA) receptors (Sheldon and Robinson, 2007). Overactivation of these receptors can result in excessive calcium influx, potentially disrupting the intracellular balance of calcium and other ions. In agreement with such observations, stimulation of EAATs appears to have neuroprotective effects against excitotoxicity through efficacious glutamate control (Masliah et al., 1998).

Currently, there are no effective therapeutic interventions for AD, with present FDA-approved drugs providing short-term efficacy at best. Further understanding of pathological changes to the glutamatergic system, in particular its homeostatic mechanisms and transporter alterations, can offer potential therapeutic targets in the future. Modulation of EAATs has also been shown to have possible therapeutic effects, with their ability to alter glutamate levels providing a logical link towards managing glutamate excitotoxicity. Upregulation of EAAT2 has been shown to reduce excitotoxic damage seen in a variety of acute and chronic neurological diseases (Sheldon and Robinson, 2007), with over-expression of EAAT3 appearing to be neuroprotective by decreasing the levels of extracellular glutamate (Lewerenz et al., 2006). Despite clear evidence of the glutamatergic system’s role in neurodegeneration (Maragos et al., 1987; Greenamyre et al., 1988; Butterfield and Pocernich, 2003; Yeung et al., 2020a,b, 2021; Kwakowsky et al., 2021), the expression of EAAT2 is yet to be explored in AD. The results reported from the few human studies are inconclusive and controversial, with hippocampal studies that lack quantitative data (Li et al., 1997; Jacob et al., 2007) or information on the region and/or layer specificity of the transporter expression within hippocampal subfields of AD and control brains (Li et al., 1997; Abdul et al., 2009).

In this study, we examined the region- and layer-specific expression and pattern changes of EAAT2 within the hippocampus, subiculum, entorhinal cortex, and superior temporal gyrus (STG) in AD post-mortem samples compared to control, to gain a better understanding of how the glutamatergic system is altered in the disease.

## Materials and Methods

### Human Brain Tissue Preparation and Neuropathological Analysis

The post-mortem human brain tissue was acquired through a donor program and was obtained from the Neurological Foundation Human Brain Bank. The procedures were approved by the University of Auckland Human Participant’s Ethics Committee (Approval number: 011654). Processing of tissue was performed as described in Waldvogel et al. (2006). The right hemisphere of the brain was fixed by perfusion with 15% formalin, cut into anatomical blocks, cryoprotected with sucrose solutions, and frozen at −80°C. Hippocampal (also containing the subiculum and entorhinal cortex) and STG blocks were used for this study. Nine control (Table 1) and eight AD cases (Table 2), with an average age of 78.5 years and a maximum post-mortem time of 48 h were used for immunohistochemistry (IHC).

**Table 1:** Normal human brain case details used for immunohistochemistry.

Case	Age	Sex	PMD	Cause of death	Weight (g)
H122	72	F	9	Emphysema	1,230
H123	78	M	7.5	Aortic aneurysm	1,260
H169	81	M	24	Asphyxia	1,225
H180	73	M	33	Ischemic heart disease	1,318
H181	78	F	20	Aortic aneurysm	1,292
H202	83	M	14	Aortic aneurysm	1,245
H226^a^	73	F	48	Mesothelioma	1,279
H239^a^	64	M	15.5	Ischemeic Heart Disease	1,529
H245	63	M	20	Asphyxia	1,194

*^a^Cases used for 3,3^′^-diaminobenzidine-peroxidase immunohistochemistry. Post-mortem delay (PMD)*.

**Table 2:** Alzheimer’s disease human brain case details used for immunohistochemistry.

Case	Age	Sex	PM delay	Cause of death	CERAD Classification	Braak and Braak Score	Weight (g)
AZ45	82	M	4.5	Pneumonia	Probable AD	IV	1,230
AZ88^a^	83	M	21	Pneumonia	Definite AD	IV	1,121
AZ90	73	M	4	Gastrointestinal hemeorrhage	Definite AD	IV	1,260
AZ92	93	F	11.5	Bronchopneumonia	Probable AD	IV	1,225
AZ98	91	F	20.5	Alzheimer’s dementia/atrial fibrillation	Definite AD	VI	1,318
AZ102	84	F	14.5	Lower respiratory tract infection	Definite AD	VI	1,292
				and hyaline arteriosclerosis
AZ103	87	M	<24	Cerebrovascular accident	Definite AD	VI	1,245
AZ113^a^	77	M	3.5	Alzheimer’s dementia/pneumonia	Definite AD	IV	1,261

*^a^Cases used for 3,3^′^-diaminobenzidine-peroxidase immunohistochemistry*.

All Alzheimer’s cases used in this study had clinical dementia. All control cases used had no history of any primary neurodegenerative, psychiatric disorder, and neurological disease abnormalities. Sections from the middle frontal gyrus, middle temporal gyrus, cingulate gyrus, hippocampus, caudate nucleus, substantia nigra, locus coeruleus, and cerebellum were examined from both control and AD groups by a neuropathologist. The distribution and density of tau and Aβ pathology were examined immunohistochemically. Based on neuritic plaque density AD cases were classified into sparse, moderate, or frequent according to the criteria from the Consortium to Establish a Registry for AD (Mirra et al., 1991), and cases that fit this criterion for definite or probable AD were included in this study.

### Western Blotting

Specificity of the primary antibodies has been tested using Western blotting (Supplementary Figure 1A) and reported previously (Simpson et al., 2010; Yao et al., 2015; Wang et al., 2017; Germany et al., 2018; Bacci et al., 2019; Castaneda-Cabral et al., 2020; Li et al., 2020; Wilkie et al., 2020; Yoshino et al., 2020). Western blotting was performed as described by Kwakowsky et al. (2018). Protein concentrations of the human hippocampal tissue samples (20 μg) were measured by using the Bio-Rad Detergent Compatible Protein assay (Bio-Rad, California, USA). Twenty microgram of each protein extract and the Precision Plus molecular weight ladder (Bio-Rad, California, USA) were run on a gradient—polyacrylamide electrophoresis gel (NU PAGE 4–12% BT 1.5, NP0336BOX; Life Technologies, Carlsbad, CA, USA) and then blotted. Proteins were separated in XCell SureLock Mini-Cell system (Invitrogen, Scoresby, VIC, Australia) and transferred onto nitrocellulose membranes using a Mini Trans-Blot Electrophoretic Transfer system (Bio-Rad, California, USA). The membranes were washed in Tris-buffered saline pH 7.6, 0.1% Tween (TBST) for 5 min and then blocked with LiCor Odyssey Blocking Buffer (LI-COR Biosciences, Nebraska, USA) for 30 min at RT. Following another 5-min wash with TBST the membranes were overnight incubated at 4°C with the EAAT2 primary antibody diluted in 4% BSA-TBST (1:500). After three 5-min TBST washes the membrane was incubated with the secondary antibody (goat anti-mouse IRDye^®^800CW, 926-32210, RRID:AB_621842) for 1 h at RT. Finally, the membrane was washed three times in TBST for 10 min each, followed by a wash in TBS for 10 min, and imaged on the Chemidoc MP Imaging System (BioRad).

### Immunohistochemistry

Coronal sections of the hippocampus, subiculum, entorhinal cortex, and STG were cut on a freezing microtome at 60 μm and stored at 4°C in phosphate-buffered saline (PBS) containing 0.1% sodium azide. Two hippocampal and two STG sections were immunostained with an EAAT2 specific antibody. The hippocampal block starts from the midpoint of the anterior commissure at +21.2 mm (containing the hippocampus, subiculum, and entorhinal cortex, plate 38–41) and the STG block at +9.3 mm (plate 29–33 according to the Mai et al. brain atlas (Mai et al., 2008). Free-floating 3,3′-diaminobenzidine (DAB)-peroxidase and fluorescent IHC (Waldvogel et al., 2006; Kwakowsky et al., 2018) were utilized for the visualization of EAAT2. All antibody dilutions were optimized. Primary antibodies and dilutions are described in Table 3. The omission of the primary antibodies resulted in a complete absence of immunoreactivity (Supplementary Figure 1B). Primary antibodies were diluted in 1% normal goat serum, and 0.04% merthiolate in PBS (immunobuffer).

**Table 3:** Primary antibodies used in this study.

Antigen	Immunogen	Source, Host, Species, Catalogue Number	Dilutions
EAAT2	Amino acids 1–85 mapping near the N terminus of EAAT2 of human origin.	Santa Cruz Biotech, Mouse, sc-365634 (E-1), RRID:AB_10844832.	1:2,000
GFAP	Full-length native protein of cow glial fibrillary acidic protein.	Abcam, Chicken, ab4674, RRID:AB_304558.	1:10,000
Anti-Neuronal Nuclei (NeuN)	Purified cell nuclei from mouse brain.	Millipore, Rabbit, ABN78, RRID:AB_10807945.	1:1,000
Anti-Neuronal Nuclei (NeuN)	GST-tagged recombinant protein corresponding to the N-terminus of mouse NeuN.	Millipore, Guinea pig, ABN90P, RRID:AB_2341095.	1:1,000

### DAB-Peroxidase Immunohistochemistry

DAB-peroxidase IHC was performed as described by Kwakowsky et al. (2018). In brief, sections were washed in PBS with 0.2% Triton X-100 (PBST) before blocking for endogenous peroxidases (50% methanol and 1% H_2_O_2_) for 20 min, followed by three 10-min washes in PBST and incubated for 72 h in primary antibody in immunobuffer at 4°C (Table 3). Following three 10-min washes in PBST the sections were incubated for 24 h with the biotinylated secondary antibody (anti-mouse IgG-Biotin antibody produced in goat 1:1,000) in immunobuffer at room temperature (RT). The sections were then washed in PBST before incubation with ExtrAvidin (1:1,000, E2886; Sigma, St. Louis, MO, USA) in immunobuffer for 4 h at RT, followed by three 10-min washes in PBST before development in 0.05% DAB and 0.01% H_2_O_2_ in 0.1 M phosphate buffer. Sections were washed in PBST, mounted onto glass slides, dried, dehydrated through a graded series of ethanol, and cleared in xylene. The slides were coverslipped with DPX mountant (1019790500; Merck, Whitehouse Station, NJ, USA). The sections were imaged on either a Leica DMRB light microscope or a Leica MZ6 dissecting microscope (Wetzlar, Germany).

### Fluorescent Immunohistochemistry

A total of 13 cases, seven control, and six AD were used in this experiment. Two hippocampal and two STG tissue sections from each case were randomized following standard simple randomization procedures in a blinded fashion. Free-floating fluorescent IHC was performed as described previously by Kwakowsky et al. (2018). In brief, sections were incubated in PBST overnight at 4°C followed by three 10-min washes with PBST and incubation for 72 h in the primary antibodies EAAT2 and NeuN and/or GFAP diluted in immunobuffer at 4°C (Table 3). Sections were washed three times for 10 min in PBST before the addition of secondary antibodies goat anti-mouse Alexa Fluor 647 (1:500, A21236, RRID:AB_141725; Invitrogen), goat anti-rabbit Alexa Fluor 488 (1:500, A11034, RRID:AB_2576217; Invitrogen), goat anti-guinea pig Alexa Fluor 594 (1:500, A11076, RRID:AB_141930; Invitrogen), goat anti-chicken Alexa Fluor 488 (1:500, ab150173, RRID:AB_2827653; Abcam), and incubated for a further 24 h at RT. Sections were then washed for 10 min in PBST before incubation for 35 min at RT with Hoechst nuclei counterstain (1:10,000, 33342, RRID:AB_10626776, Invitrogen) diluted in PBS. After three subsequent 10-min washes in PBS, sections were mounted onto glass slides, coverslipped with Mowiol mounting medium, and sealed with nail varnish.

### Imaging and Analysis

Imaging was conducted using a Zeiss 710 inverted confocal laser-scanning microscope (Carl Zeiss, Jena, Germany). Brain regions and layers were differentiated based on cell type and relative location, utilizing NeuN and Hoechst staining. An argon laser was used to excite NeuN-positive neurons at a 488-nm wavelength, a helium-neon laser with a 633 nm wavelength was used for Alexa 647 immunolabeled antigens of interest, and a blue diode laser with a 405 nm wavelength was used for Alexa 405 for Hoechst counterstained nuclei with a 20x objective. After background subtraction and grayscale threshold determination, EAAT2 density measurements were performed from a 31,000 μm^2^ area in each analyzed layer in the dentate gyrus (DG; str. granulosum, str. moleculare and hilus), CA1, CA2, and CA3 subregions (str. oriens, str. pyramidale, str. radiatum) using ImageJ software (U. S. National Institutes of Health, Bethesda, Maryland, USA). Density measurements for the subsequent regions were obtained from a 432,000 μm^2^ region in the subiculum, a 605,000 μm^2^ region in the entorhinal cortex, and a 692,000 μm^2^ region in the STG through all cortical layers. Both the threshold and the size of the region of interest were constant across all sections for each region in each experiment. The image acquisition and analysis were performed blinded to eliminate bias during the experiment.

### Statistical Analysis

The data did not meet the assumptions of parametric tests assessed by the D’Agostino–Pearson omnibus and Brown–Forsythe tests. Therefore, to examine differences between groups, an unpaired Mann–Whitney test was used. No data points were identified and excluded as outliers using the ROUT method. All statistical analyses were conducted using Graph-Pad Prism software version 8 (GraphPad software; RRID:SCR_002798) with a value of *p* ≤ 0.05 considered significant. Adobe Photoshop CC 2018 (Adobe Systems Software, San Jose, CA, USA) was used to prepare the figures. All experimental data are expressed as the mean ± Standard Error of Mean (SEM).

## Results

### Expression of EAAT2 in the Human Hippocampus, Subiculum, Entorhinal Cortex, and Superior Temporal Gyrus

**Figure 1:**
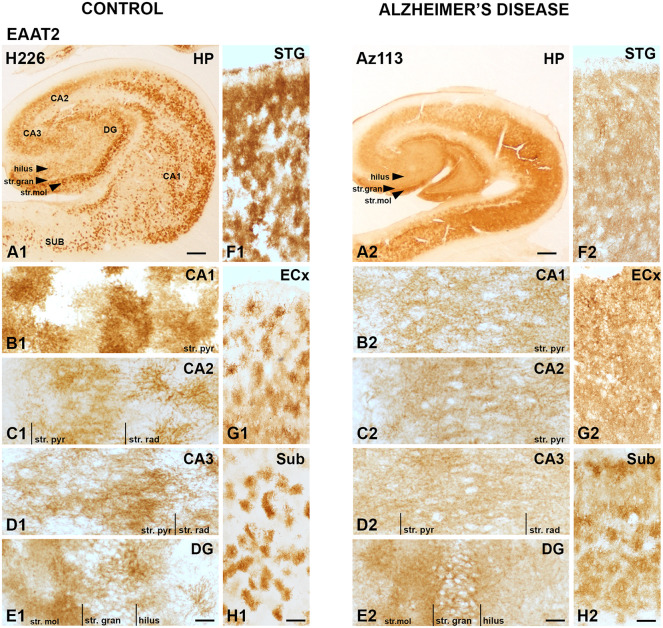
EAAT2 expression in the hippocampus, subiculum, entorhinal cortex, and superior temporal gyrus in human control and Alzheimer’s disease (AD) cases visualized by 3,3′-diaminobenzidine-peroxidase immunohistochemistry. The EAAT2 staining is localized to astrocytes and appears relatively strong within the str. pyramidale of the CA1 subregion **(A1,B1)**, the stratum (str.) moleculare of the dentate gyrus **(A1,E1)** with more diffuse labeling in AD cases **(A2–H2)**. CA, cornu ammonis; DG, dentate gyrus; ECx, entorhinal cortex; HP, hippocampus; STG, superior temporal gyrus; str. pyr, stratum pyramidale; str. rad, stratum radiatum; str. gran, stratum granulosum; Sub, subiculum. Scale bars: **(A1–A2)** = 1,000 μm; **(B1–E1, B2–E2)** = 100 μm; **(F1–H1, F2–H2)** = 400 μm.

**Figure 2:**
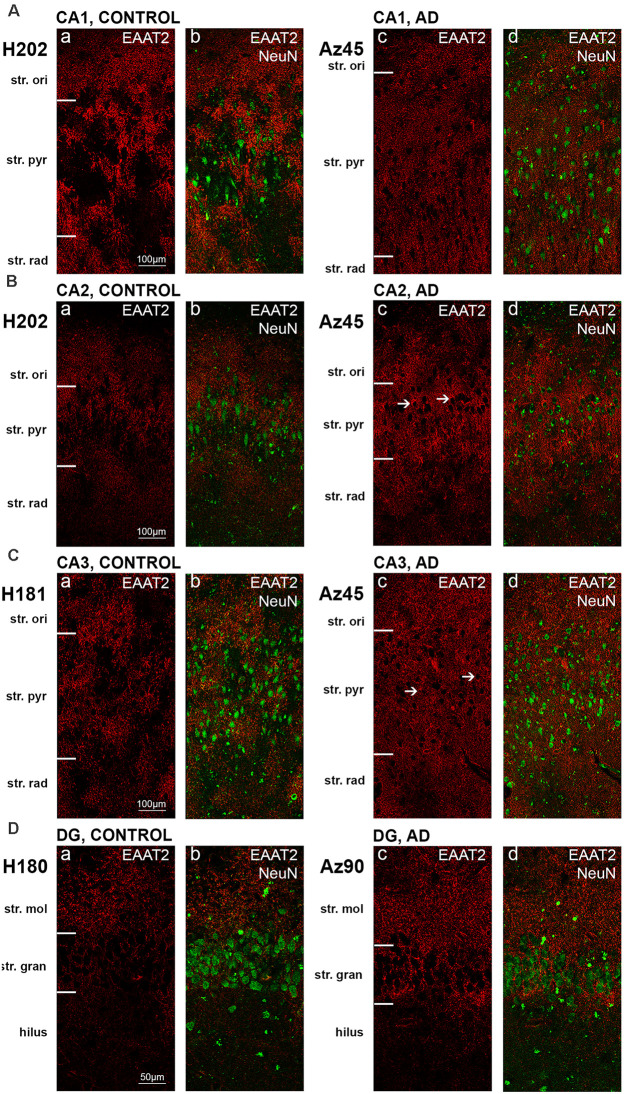
EAAT2 expression in the hippocampus in human control and Alzheimer’s disease (AD) cases visualized by fluorescent immunohistochemistry. Photomicrographs of representative regions of the CA1 **(A)**, CA2 **(B)**, CA3 **(C)**, and dentate gyrus **(D)** showing EAAT2 (red) and EAAT2 overlaid with NeuN (green) immunoreactivity for representative AD and control cases. AD, Alzheimer’s disease; CA, cornu ammonis; DG, dentate gyrus; str. ori, straum oriens; str. pyr, stratum pyramidale; str. rad, stratum radiatum; str. mol, stratum moleculare; str. gran, stratum granulosum. Scale bars **(A–C)** = 100 μm; **(D)** = 50 μm.

**Figure 3:**
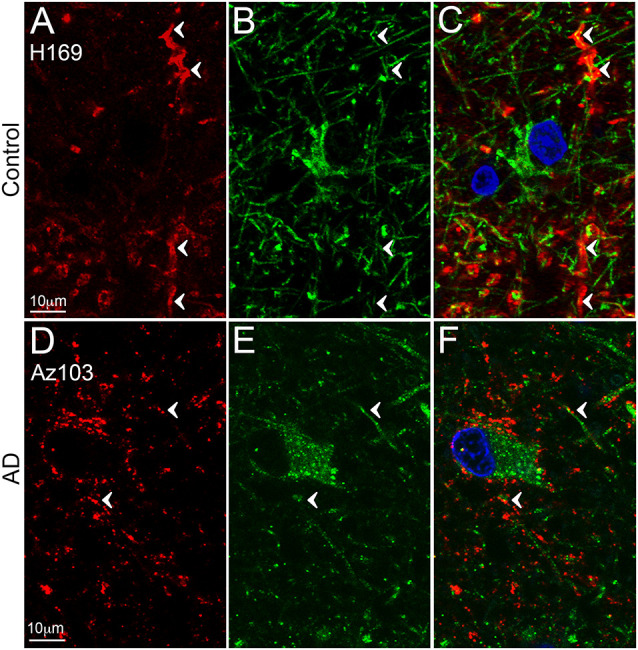
EAAT2 expression in the dentate gyrus. Photomicrographs of the stratum moleculare of the denate gyrus showing EAAT2 (**A,D**, red), GFAP (**B,E**, green) and EAAT2 overlaid with GFAP **(C,F)** immunoreactivity for a representative control (**A,B**, H169) and Alzheimer’s disease (AD) (**D–F**, Az103) case. EAAT2 labeling is mainly localized to membranes of astrocytic stem and fine branches, and soma, while GFAP staining is observed in the cytoplasm of the branches and soma (**A–F**, arrowheads). Cell nuclei are labeled with Hoechst nuclei counterstain (**C,F**, blue). Scale bar **(A–C)** = 10 μm.

**Figure 4:**
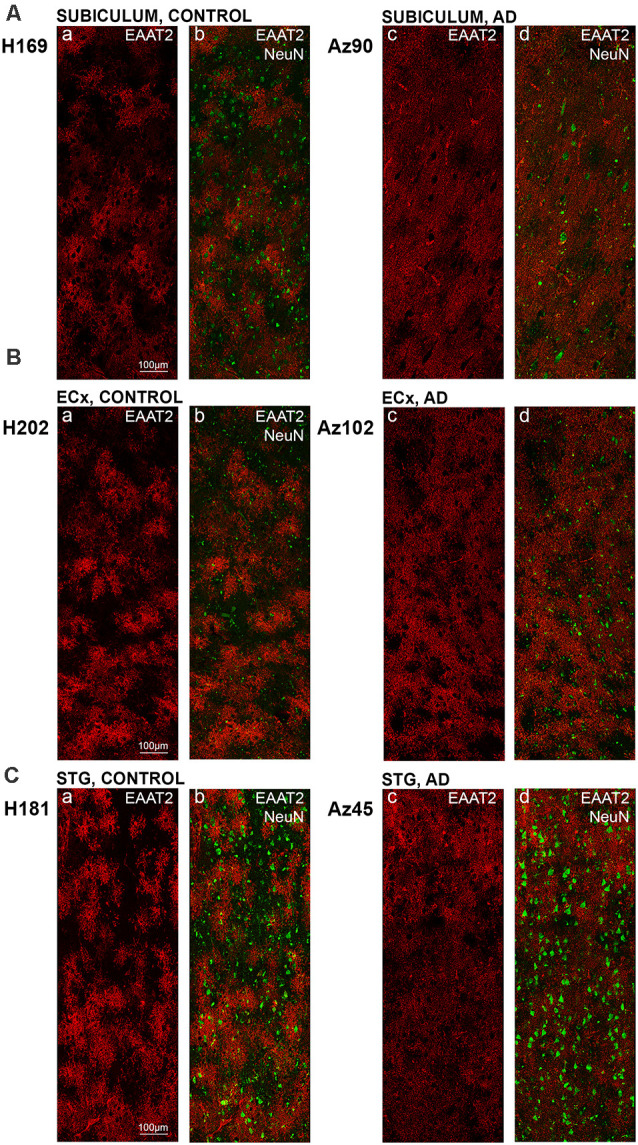
EAAT2 expression in the subiculum, entorhinal cortex, and superior temporal gyrus in human control and Alzheimer’s disease (AD) cases. Photomicrographs of representative regions of the subiculum **(A)**, entorhinal cortex **(B)**, and STG **(C)** showing EAAT2 (red) and EAAT2 overlaid with NeuN (green) immunoreactivity for representative AD and control cases. AD, Alzheimer’s disease; CA, cornu ammonis; DG, dentate gyrus; ECx, entorhinal cortex: STG, superior temporal gyrus. Scale bars **(A–C)** = 100 μm.

**Figure 5:**
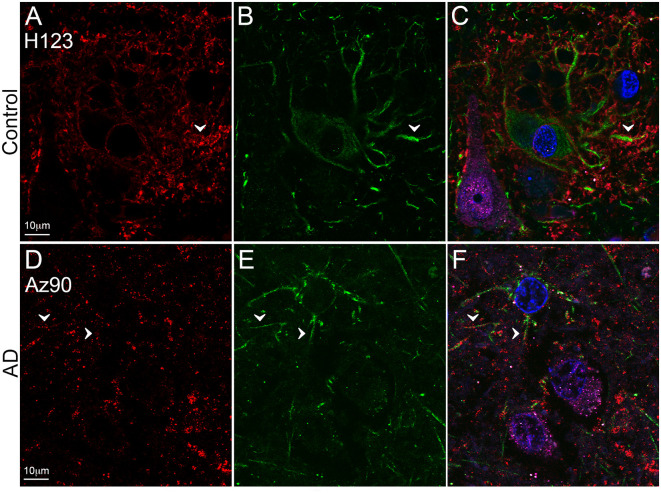
EAAT2 expression in the superior temporal gyrus (STG). Photomicrographs of layer III of the STG showing EAAT2 (**A,D**, red), GFAP (**B,E**, green), and EAAT2 overlaid with GFAP and NeuN (magenta; **C,F**) immunoreactivity for a representative control (**A–C**, H123) and Alzheimer’s disease (AD) (**D–F**, Az90) case. In controls, EAAT2 labeling is more localized to membranes of astrocytic stem processes, while in AD the staining is weaker on astrocytic main branches (**A–F**, arrowheads), but more labeling is likely localized to fine astrocytic branches in the neuropil. Cell nuclei are labeled with Hoechst nuclei counterstain (**C,F**, blue). Scale bar **(A–F)** = 10 μm.

EAAT2 DAB IHC revealed strong astrocytic staining across all brain regions examined, with particularly strong staining in the CA1 subfield (Figures 1A1,B1), the str. moleculare of the DG (Figures 1A1,E1), and the STG (Figure 1F). Both control and AD cases display strong immunoreactivity in astrocytes, which confirms literature indicating its expression in astrocytes, but the AD cases show more labeling in the neuropil that makes individual astrocytes less recognizable (Figure 1). This is better visualized with fluorescence IHC (Figures 2–5).

Astrocytic staining was patchy in some regions, but much more intense and condensed in others. Within the CA1 subfield, immunoreactivity was localized to astrocytic processes and appeared diffuse throughout the str. pyramidale, str. oriens, and str. radiatum (Figure 2A). There appears to be a lack of staining surrounding and within neuronal bodies stained with NeuN, with the majority of labeling observed on astrocytic processes (Figure 2A). Within the CA2 subfield, EAAT2 immunolabeling was relatively uniform between the three layers, with slightly higher expression levels within the str. pyramidale (Figure 2B). Staining appears to be localized only to astrocytes and not neurons. In comparison to control sections, the CA2 region of AD sections exhibited much stronger immunoreactivity in astrocytic main branches surrounding some of the NeuN positive cell bodies within the str. pyramidale (Figure 2B, arrows), and this difference was also observed within the CA3 (Figure 2C, arrows) and the str. granulosum of the DG (Figure 2D). The CA3 subfield and DG exhibited a similar staining pattern to the CA1 and CA2 (Figures 2C,D). Within the DG, there was greater immunoreactivity in the str. moleculare compared to the hilus and the str. granulosum (Figure 2D). The subiculum (Figure 4A), entorhinal cortex (Figure 4B), and STG (Figure 4C) regions exhibited similar staining patterns, with immunolabeling on astrocytes and on their processes, and AD cases displaying much more diffuse staining (Figures 4A–C, 5A–F). Protoplasmic astrocytes between layers II-VI of the entorhinal cortex and STG (Oberheim et al., 2009) are strongly stained for EAAT2 and this results in patchy staining. Interlaminar astrocytes in cortical layer I and polarized astrocytes in cortical layers V to VI also express EAAT2. Protoplasmic astrocytes often overlap with one another and also show overlap with polarized astrocytes (Figure 4; Oberheim et al., 2009).

**Figure 6:**
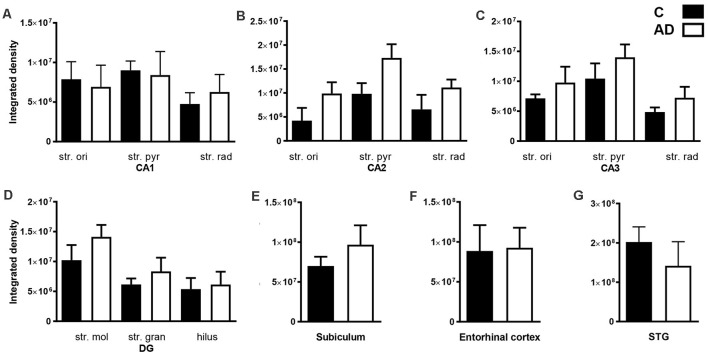
Quantification of EAAT2 immunoreactivity within the CA1, CA2, CA3, dentate gyrus hippocampal subfields, subiculum, entorhinal cortex, and STG in control and AD groups. In the hippocampal CA1 **(A)**, CA2 **(B)**, CA3 **(C)**, DG **(D)**, subiculum **(E)**, entorhinal cortex **(F)** and STG **(G)** EAAT2 density shows no statistically significant change in AD (white bars; *n* = 6) compared to control (black bars; *n* = 7) cases (Unpaired Mann–Whitney test). Data are expressed as mean with error bars representing standard error of mean (SEM). AD, Alzheimer’s disease; C, control; CA, cornu ammonis; DG, dentate gyrus; STG, superior temporal gyrus, str. ori, stratum oriens; str. pyr, stratum pyramidale; str. rad, stratum radiatum; str. mol, stratum moleculare; str. gran, stratum granulosum.

Quantification of EAAT2 labeling density performed based on fluorescence IHC experiments (Figures 2, 4) did not reveal any statistically significant differences between AD and control brains in any of the brain regions investigated (Figures 6A–G). While the labeling can be stronger on some individual astrocytes in controls, this does not result in overall transporter density differences between control and AD due to the increased expression in neuropil in AD cases.

## Discussion

The present study is the first to provide a comprehensive examination of expression levels and patterns of glutamate EAAT2 in the human hippocampus, subiculum, entorhinal cortex, and STG, and how this expression is altered in AD. EAAT2 shows strong labeling of astrocytic cell bodies and processes in all these brain regions. We report a significantly altered staining pattern of EAAT2 in AD cases, with a more diffuse staining in the neuropil, particularly within the CA1–3 and DG regions. Our findings indicate no significant region- and layer-specific density changes of this glutamate transporter in the human hippocampus, subiculum, entorhinal cortex, and STG in comparison to healthy controls.

EAAT2 is mainly expressed in astrocytes and is responsible for ~95% of all L-glutamate uptake in the CNS from the synaptic cleft (Vandenberg and Ryan, 2013). EAAT2 is also an important element of the glutamate-glutamine cycle (Beart and O’Shea, 2007). During the past few decades, mouse models have provided important but conflicting data regarding the role and regulation of glutamate transporters in AD pathology. However, AD is a human disease, and studies involving human tissue remain the most reliable and representative mode of investigating pathological changes. Human studies involving EAAT2 expression changes have however been scarce. An early radiolabeling study found a ~30% decrease in [^3^H]aspartate binding in the midfrontal cortex of AD brains, suggesting decreased glutamate transporter activity associated with increased excitotoxicity and neurodegeneration (Masliah et al., 1996). EAAT2 IHC revealed strong glial labeling in the frontal cortex and hippocampus with reduced astrocytic localization in AD cases, but no quantitative data was provided regarding the hippocampal expression (Li et al., 1997). Interestingly, the pattern of the staining in the hippocampus (the hippocampal subfield is not identified) seems to be similar to our findings, with an increased labeling in the neuropil (Li et al., 1997). Jacob et al. (2007) reported impairment in the expression of EAAT1 and EAAT2 at both gene and protein levels in the hippocampus and gyrus frontalis medialis of AD patients, but up-regulation in the cerebellum. These findings confirm that EAAT2 expression alterations are brain region-specific in AD. However, the semi-quantitative examination by Jacob et al. (2007) does not show a clear decrease in EAAT2 expression in the hippocampus. The low case number (*n* = 4, controls), and high variability in staining pattern and intensity make it difficult to draw any significant conclusions. Furthermore, the immunohistochemical labeling of EAAT2 is relatively weak and the differences between control and AD cases are not shown. EAAT2 is the major glutamate transporter and represents 1% of total brain protein, therefore EAAT2 labeling would be expected to be strong and widespread in the hippocampus (Lehre and Danbolt, 1998). In contrast, other studies have demonstrated no decrease in the expression of EAAT2 in AD. In the cingulate and inferior temporal gyri, EAAT2 protein levels are well preserved in AD subjects, with normal transporter levels found in a high percentage of AD cases (Beckstrom et al., 1999). EAAT2 expression was also preserved in the frontal cortex in the advanced stages of AD (Garcia-Esparcia et al., 2018). Our study, the first comprehensive examination of EAAT2 expression in the AD hippocampus, subiculum, entorhinal cortex, and STG, does not show EAAT2 density change in AD either.

EAAT2 is predominantly expressed in astrocytes, although they are also expressed in other types of glial cells, including microglia, macrophages, and oligodendrocytes (Kondo et al., 1995; Gras et al., 2012; Parkin et al., 2018; Pajarillo et al., 2019). Whilst astrocytic EAAT2 staining is well established, the presence of EAAT2 in neurons is controversial. Neuronal EAAT2 mRNA expression has been demonstrated in multiple rat studies (Torp et al., 1994; Schmitt et al., 1996; Berger et al., 2005) but its presence at the protein level is still controversial. Multiple animal and few human studies suggest that EAAT2 protein is exclusively expressed in astrocytes (Rothstein et al., 1994; Lehre et al., 1995; Li et al., 1997; Simpson et al., 2010) while others provide evidence of neuronal expression (Rimmele and Rosenberg, 2016). However, several technical issues could lead to false-positive findings, such as weak antibody labeling that might represent nonspecific background staining; labeling that is localized to astrocytic processes wrapping around the neurons rather than the expression on neuronal membranes; and astrocytic contamination of synaptosomes (Rimmele and Rosenberg, 2016). Differences in tissue processing methodology can also contribute to variable findings regarding EAAT2 protein localization and expression levels. Interestingly, one study reported a large variability in astrocytic EAAT2 expression in AD human tissue within the lateral temporal cortex, and categorized the cases into three groups with minimal, moderate, or extensive immunoreactivity (Simpson et al., 2010). However, while the number of astrocytes and their morphology were variable between cases, we did not observe the “minimal and extensive” type of staining that might represent the lack of staining or high non-specific background labeling. While the authors ruled out that neither pH nor post-mortem delay (PMD) significantly correlates with either GFAP or EAAT2 immunoreactivity, they were not able to exclude the effect of fixation on the detection of these proteins. Long–term storage in formalin can significantly influence antibody binding, therefore our protocol involves a standardized fixation protocol followed by cryoprotection with sucrose solutions, freeze down, and storage at −80°C. However, staining variability is one of the main challenges of using human tissue, which can be the result of many other factors related to post-mortem conditions that cannot be controlled or correlated with transporter expression patterns. In this study, we did not observe neuronal EAAT2 expression, but strong labeling is localized to astrocytic processes wrapping around the neurons.

Reactive astrocytes are easily identified by their GFAP immunoreactivity, but GFAP expression in non-reactive (resting) astrocytes is often below the detection level of IHC, that can make co-localization experiments challenging. The up-regulation of GFAP in reactive astrocytes can impair physiological protein degradation and restrict migration and process motion (Orre et al., 2013; Perez-Nievas and Serrano-Pozo, 2018). With significant neuronal loss, the reorientation of astrocyte processes towards amyloid plaques, and increased astrocytosis, the cellular architecture of the hippocampus and cortex seems to be disorganized in AD compared to healthy controls (Buldyrev et al., 2000; Colombo et al., 2000, 2002), which might contribute to the diffuse EAAT2 staining that remains localized to astrocytic processes. Furthermore, in AD, more EAAT2 staining seems to localize to neuropil; this could be the result of labeling on fine astrocytic branches that are usually not stained with GFAP (Derouiche and Frotscher, 2001). However, it is also possible that the staining is localized to fragmented astrocyte processes and this can result in staining that appears as homogenous labeling of the neuropil.

In conclusion, no significant EAAT2 density changes were found between control and AD cases, however, the observed spatial differences in transporter expression could underly alterations in glutamate recycling and potentially disturbed glutamatergic homeostasis. Further studies will be required to explore how EAAT2 function is affected in AD and its potential as a therapeutic target.

## Data Availability Statement

The original contributions presented in the study are included in the article, further inquiries can be directed to the corresponding author.

## Ethics Statement

The studies involving human participants were reviewed and approved by University of Auckland Human Participant’s Ethics Committee. The patients/participants provided their written informed consent to participate in this study.

## Author Contributions

JY, TP, OW, HW, and AK performed the research. CT carried out the pathology for all human brain tissue. AK, RF, and HW designed the research. JY and AK wrote the manuscript. AK was responsible for project administration. HW, RF, and AK supervised the study. All authors contributed to the article and approved the submitted version.

## Conflict of Interest

The authors declare that the research was conducted in the absence of any commercial or financial relationships that could be construed as a potential conflict of interest.

## Publisher’s Note

All claims expressed in this article are solely those of the authors and do not necessarily represent those of their affiliated organizations, or those of the publisher, the editors and the reviewers. Any product that may be evaluated in this article, or claim that may be made by its manufacturer, is not guaranteed or endorsed by the publisher.

## Supplementary Material

SUPPLEMENTARY FIGURE 1 Western blot against human hippocampus homogenates probed with EAAT2 antibody **(A)**. The omission of the primary antibodies **(B)** resulted in a complete absence of immunoreactivity except for a small amount of background lipofuscin staining **(B,c)**. The section was stained with goat anti-mouse Alexa Fluor 647 **(B,b)** and goat anti-rabbit Alexa Fluor 488 **(B,c)**. Nuclei were counterstained with Hoechst dye (blue; **B,a**). Scale bar B = 25 μm.

## Abbreviations

Aβ_1–42_: Amyloid Beta
AD: Alzheimer’s Disease
CA: Conus Ammonis
CNS: central nervous system
DG: Dentate Gyrus
EAAT2: Excitatory amino acid transporter 2
GABA: γ-aminobutyric acid
GFAP: Glial fibrillary acidic protein
NMDA: N-methyl-D-aspartate
PBS: phosphate-buffered saline
PMD: Post-mortem delay
RRID: Research Resource Identifier
RT: Room Temperature
STG: Superior temporal gyrus
Str: Stratum
TBS: Tris-Buffered Saline
TBST: Tris-Buffered Saline with Tween.
