# Supplementary figures and images for: EAAT2 Expression in the Hippocampus, Subiculum, Entorhinal Cortex and Superior Temporal Gyrus in Alzheimer’s Disease
Source: Front Cell Neurosci. 2021 Sep 13;15:702824. doi: 10.3389/fncel.2021.702824 (PMC8475191; doi:10.3389/fncel.2021.702824)

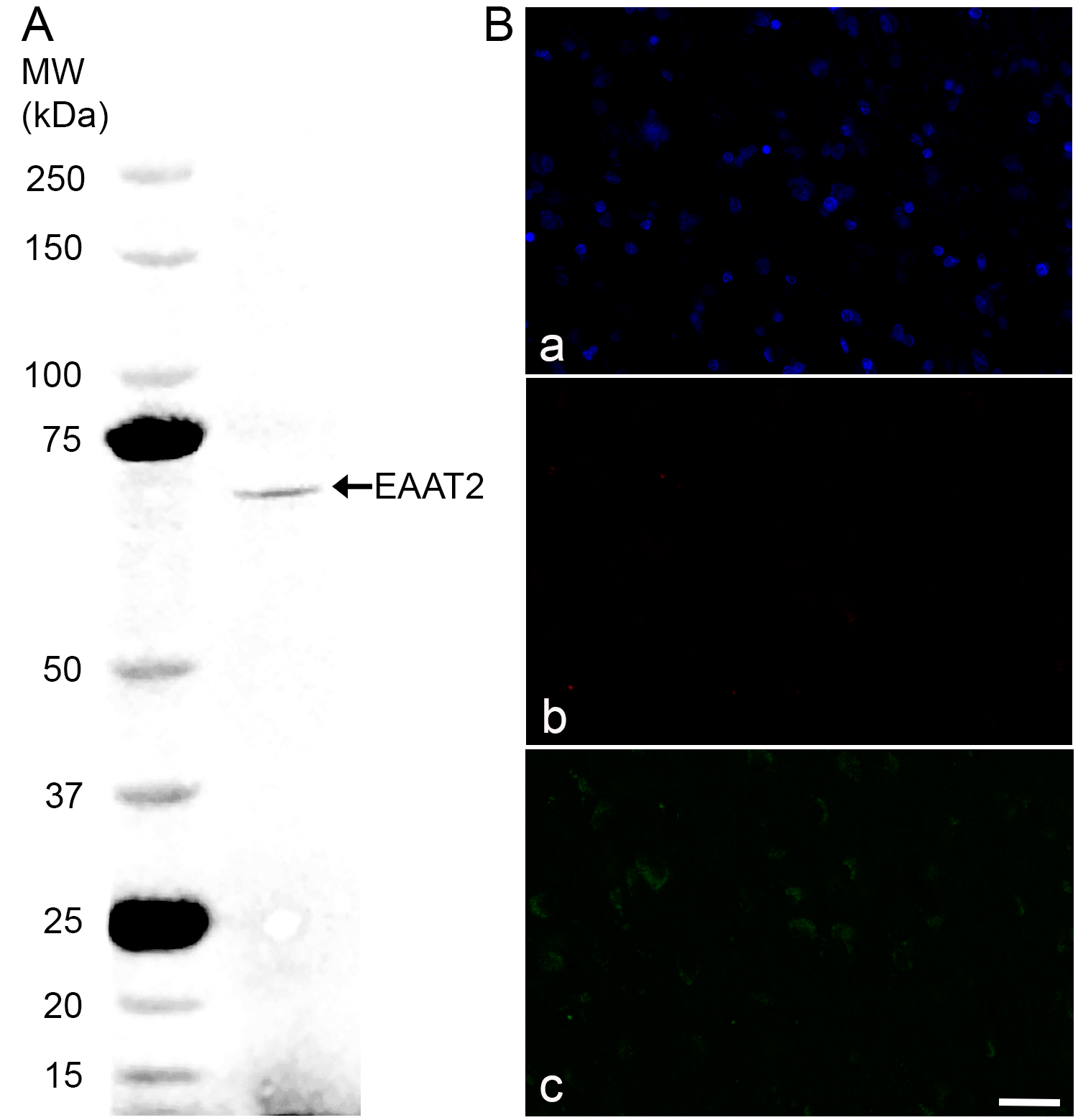

Supplement: SUPPLEMENTARY FIGURE 1 — Western blot against human hippocampus homogenates probed with EAAT2 antibody (A). The omission of the primary antibodies (B) resulted in a complete absence of immunoreactivity except for a small amount of background lipofuscin staining (B,c). The section was stained with goat anti-mouse Alexa Fluor 647 (B,b) and goat anti-rabbit Alexa Fluor 488 (B,c). Nuclei were counterstained with Hoechst dye (blue; B,a). Scale bar B = 25 μm. [file Image_1.TIF]
